# Involvement of Galectin-9/TIM-3 Pathway in the Systemic Inflammatory Response in Early-Onset Preeclampsia

**DOI:** 10.1371/journal.pone.0071811

**Published:** 2013-08-02

**Authors:** Eva Miko, Matyas Meggyes, Barbara Bogar, Nora Schmitz, Aliz Barakonyi, Akos Varnagy, Balint Farkas, Peter Tamas, Jozsef Bodis, Julia Szekeres-Bartho, Zsolt Illes, Laszlo Szereday

**Affiliations:** 1 Department of Medical Microbiology and Immunology, Clinical Centre, University of Pecs, Pecs, Hungary; 2 Department of Obstetrics and Gynaecology, Clinical Centre, University of Pecs, Pecs, Hungary; 3 Department of Neurology, Odense University Hospital, Odense, Denmark; 4 Institute of Clinical Research, University of Southern Denmark, Odense, Denmark; 5 Janos Szentagothai Research Centre, Pecs, Hungary; VU University Medical Center, Netherlands

## Abstract

**Background:**

Preeclampsia is a common obstetrical disease affecting 3-5% of pregnancies and representing one of the leading causes of both maternal and fetal mortality. Maternal symptoms occur as an excessive systemic inflammatory reaction in response to the placental factors released by the oxidatively stressed and functional impaired placenta. The T-cell immunoglobulin domain and mucin domain (TIM) family is a relatively newly described group of molecules with a conserved structure and important immunological functions. Identification of Galectin-9 as a ligand for TIM-3 has established the Galectin-9/TIM-3 pathway as an important regulator of Th1 immunity and tolerance induction.

**Methods:**

The aim of our study was to investigate the expression and function of Galectin-9 and TIM-3 molecules by peripheral blood mononuclear cells and the possible role of Galectin-9/TIM-3 pathway in the immunoregulation of healthy pregnancy and early-onset preeclampsia. We determined TIM-3 and Gal-9 expression and cytotoxicicty of peripheral lymphocytes of early-onset preeclamptic women and healthy pregnant woman using flow cytometry.

**Results:**

Investigating peripheral lymphocytes of women with early-onset preeclampsia, our results showed a decreased TIM-3 expression by T cells, cytotoxic T cells, NK cells and CD56^dim^ NK cells compared to healthy pregnant women. Interestingly, we found a notably increased frequency of Galectin-9 positive cells in each investigated lymphocyte population in the case of early-onset preeclamptic patients. We further demonstrated increased cytotoxic activity by cytotoxic T and CD56^dim^ NK cells in women with early-onset preeclampsia. Our findings showed that the strongest cellular cytotoxic response of lymphocytes occurred in the TIM-3 positive subpopulations of different lymphocytes subsets in early-onset preeclampsia.

**Conclusion:**

These data suggest that Gal-9/TIM-3 pathway could play an important role in the immune regulation during pregnancy and the altered Galectin-9 and TIM-3 expression could result an enhanced systemic inflammatory response including the activation of Th1 lymphocytes in preeclampsia.

## Introduction

Preeclampsia is a common obstetrical disorder of placental origin with both local and systemic anomalies which is unique to human. It affects about 3-5% of pregnancies representing the leading cause of maternal, fetal and neonatal mortality and morbidity worldwide [1,2]. It is usually manifested in the second half of pregnancy with a classical triad of maternal symptoms: hypertension, proteinuria and edema [3]. Although the diagnosis is based on these late clinical findings, preeclampsia is thought to be an implantation disorder.

The actual hypothesis regarding the etiology of preeclampsia centers inadequate trophoblast invasion and placentation presumably as a result of maladaptation of maternal immune responses locally [4,5]. The immunological recognition of the fetus and its subsequent immunotolerance by the maternal immune system is not only the question of rejection or acceptance but it also plays a central role in implantation and placentation. Polymorphic paternal antigens expressed by extravillous cytotrophoblast provoke an inflammatory response in the decidua leading to the loosening of the tissue and facilitating trophoblast invasion and spiral artery remodeling. The recognition of monomorphic paternal antigens will limit the depth of placentation by activating local immunotolerance mechanisms of the mother [6,7]. In the case of preeclampsia, the invading trophoblast becomes excessively inhibited from the beginning on resulting in poor placentation and in a small sized placental mass [8–10].

The small placenta decompensate continuously when fetal growth is accelerated (usually from week 20 on) and maternal symptoms occur resulting from intrinsic factors (syncytiotrophoblast microvesicles-STBM, anti-angiogenetic factors, sFlt-1) released by the hypoxic and oxidatively stressed placenta into the systemic circulation [11–14].

In the background of the clinical manifestation of the disease there is a generalized systemic inflammatory response and an endothelial dysfunction [15]. Circulating STBM act pro-inflammatory and induce the secretion of TNF-α [16,17], IL-6 [16,18], IFN-γ [19] leading to the development of Th1 type immunity [20].

In earlier works, our group demonstrated the involvement of the innate immunity in the pathogenesis of the inflammatory stage of the disease, showing that in preeclampsia, peripheral γδ T cells and invariant NKT cells display an increased cytotoxic potential, which may be due to altered expression of NK cell inhibitory and activating receptors [21,22].

According to the current concept, preeclampsia is subdivided in early (before 34 weeks) and late onset (after 34 weeks) preeclampsia [4]. The major difference between the two clinical forms is the etiological role of poor placentation. The early onset type is considered to represent the “real preeclampsia” with the pathomechanism described above. Late onset preeclamptic patients have diseases/conditions like diabetes mellitus, anemia, altitude sickness or multiple pregnancies, where the placenta compensatory enlarges due to maternal hypoxia and microvascular diseases [4].

The T-cell immunoglobulin and mucin domain (TIM) family is a relatively newly described group of molecules with a conserved structure and important immunological functions [23,24]. A growing body of evidence supports the critical role of different TIM molecules as modulators of the immune response in transplant tolerance [25–27]. TIM-3 is a type I transmembrane protein that contains no defined signaling motifs in its cytoplasmic domain, but it has been implicated both in activation and inhibition of immune responses [28,29]. TIM-3 is expressed in a variety of immune cells, including CD8+ T cells [30], NK cells [31], NKT cells, Th17 cells, regulatory T cells, dendritic cells, monocytes, macrophages and mast cells. Therefore, there is mounting evidence that TIM-3 is a potent regulator of both the adaptive and innate immune response.

Identification of Galectin-9 (Gal-9) as a ligand for TIM-3 has established the Gal-9/TIM-3 pathway as an important regulator of Th1 immunity and tolerance induction [27,32,33]. Engagement of TIM-3 by its ligand Gal-9 negatively regulates IFN-γ secretion, influences the ability to induce T cell tolerance and triggers a significant signal cascade to induce apoptosis of Th1 type immune cells. Ndhlovu et al. and others observed that increased amounts of TIM-3 on T cells during HIV, hepatitis C virus, and other chronic viral infections correlated with T-cell dysfunction, suggesting that TIM-3 is also part of a regulatory pathway during T cell exhaustion [34–36].

Although data about the role of Gal-9/TIM-3 pathway in the pathogenesis of human diseases is emerging, data about their role during human pregnancy and feto-maternal immunological relationship is scarce. Taken the fact that in the second, clinical stage of preeclampsia a maternal systemic inflammatory response develops we can assume that the immunoregulatory systems fail to control inflammation and maintain the healthy immunological balance. As a part of it, the Gal-9/TIM-3 pathway may also be affected. Therefore, the aim of our present study was to investigate the expression of Gal-9 and TIM-3 molecules by peripheral blood mononuclear cells in healthy pregnancy and preeclampsia.

## Materials and Methods

### Ethics Statement

Written informed consent was obtained from all participants. The study protocol conforms to the ethical guidelines of the 1975 Declaration of Helsinki as reflected in a priori approval by the Regional Ethical Committee at the Faculty of Medicine, University of Pécs.

### Patients

27 women with the classical symptoms of early-onset preeclampsia (hypertension, proteinuria and edema) were included in the study. Hypertension was defined as a diastolic blood pressure of ≥90 mmHg on two separate occasions within a 24-h period, and proteinuria was defined by ≥0,3 g protein in 24-h urine collection. 25 healthy pregnant women appropriately matched for gestational age formed the control group (Table 1).

**Table 1:** Patients’ demographic and gynecological characteristics.

	**Healthy pregnant women**	**Early-onset preeclamptic women**	P-value
No. of patients	25	27	
Age (years) (mean)	32,6±1,03	29,1±1,53	NS
Gestational age at sampling (mean)	35,64±0,27	33,74±0,48	NS
Gestational age at birth (mean)	38,9±0,27	34,1±0,64	P<0,05
Birth weight (mean)	3420±126,84	1881±148,28	P<0,05
Previous live birth/ patients	058±0,15	088±0,31	NS

Statistical comparisons were made by using the Student’s t-tests. The results were expressed as the mean value±standard error of the mean (SEM). Differences were considered significant when the value of P was equal to or less than 0.05. NS = not statistically significant.

### Lymphocyte separation, cryopreservation and thawing

Peripheral blood mononuclear cells (PBMC) were separated from heparinized venous blood on Ficoll-Paque gradient. After washing the cells in RPMI 1640 medium the cells were counted and centrifuged.

Resuspension was performed in human serum containing 10% DMSO for cryoprotection. Cells were aliquoted in cryovials and stored in a -80^o^C mechanical freezer. Thawing was carried out on the day of fluorescent cell labeling. Cryovials were warmed up as quickly as possible in 37^o^C water bath and DMSO was washed out twice in RPMI 1640 medium.

### Antibodies

Freshly thawed PBMC were used for surface and intracellular staining and analysis. The following monoclonal antibodies were used: fluorescein isothiocyanate (FITC)-conjugated anti-human CD3 (BD Pharmingen), FITC-conjugated anti-human CD4 (BD Pharmingen), FITC-conjugated anti-human CD107a (BD Pharmingen), phycoerythrin (PE)-conjugated anti-human Galectin-9 (Biolegend), PE-conjugated anti-human TIM-3 (R&D Systems), allophycocyanin (APC)-conjugated anti-human CD56 (BD Pharmingen), APC-conjugated anti-human CD8 (BD Pharmingen), APC-conjugated anti-human FoxP3 (eBioscience). Control antibodies included isotype-matched FITC-conjugated, PE-conjugated and APC-conjugated mouse antibodies (all from BD-Pharmingen).

### Peripheral blood lymphocyte populations detected by flow cytometry

T cells (CD3+ cells), helper T cells (CD3+/CD4+), cytotoxic T cells CD3+/CD8+ cells), regulatory T cells (CD4+/FoxP3+ cells), NK cells (CD3-/CD56+ cells), CD56^dim^ NK cells (CD3-/CD56^dim^+ cells), CD56^bright^ NK cells (CD3-/CD56^bright^+ cells), and NKT cells (CD3+/CD56+ cells).

### Labeling of lymphocytes and flow cytometric analysis

10^6^ thawed PBMC in 100 μl PBS/tube was incubated for 30 minutes at room temperature with the fluorochrome-labeled monoclonal antibodies. Finally, the cells were resuspended in 300 μl PBS containing 1% paraformaldehyde, and stored at 4^o^C in dark until FACS analysis.

Labeled cells were analyzed with a FACSCalibur flow cytometer (BD Immunocytometry Systems, Erembodegen, Belgium) equipped with the CellQuest software program (BD Biosciences, San Diego, CA, USA) for data acquisition and analysis.

### CD107a functional assay

To determine CD107a surface expression by cytotoxic T cells and NK cells, PBMC were incubated for 4 h at 37° C in the presence of FITC-conjugated anti-human CD107a monoclonal antibody in RPMI 1640 medium containing 10% fetal bovine serum, penicillin and streptomycin, ionomycin (Sigma–Aldrich) and phorbol myristate acetate (Sigma–Aldrich). After stimulation the cells were washed and resuspended in PBS then stained with antibodies to cytotoxic T cell and NK cell markers (APC-conjugated anti-human CD8 or APC-conjugated anti-human CD56) together with PE-conjugated anti-human TIM-3 antibody for 30 min at room temperature at dark. The cells were washed in PBS, fixed with 1% paraformaldehyde and evaluated by FACS.

### FoxP3 staining

After surface labeling intracellular staining of Foxp3 was performed using the FoxP3 Staining Buffer Set (eBioscience) according to the manufacture’s protocol. Briefly, cells were permeabilized in 1 ml fixation/permeabilization buffer (Concentrate/Diluent 1:4) at 4° C for 1 h. Washed twice in buffer and stained afterwards with the anti-human FoxP3 monoclonal antibody at 4° C for 1 hour. Flow cytometric analysis was performed on a FACSCalibur flow cytometer (Becton Dickinson).

### Statistical analysis

Statistical comparisons were made by using the Student’s t-tests. The results were expressed as the mean ± SEM. Differences were considered significant for P ≤ 0.05.

## Results

### 1: Phenotype analysis of peripheral blood mononuclear cells in women with early-onset preeclampsia and in healthy pregnant women

As shown in Table 2 we compared the frequency of T cells, helper and cytotoxic T cell subpopulations, regulatory T cells, NK cells, CD56^dim^ NK cells, CD56^bright^ NK cells, and NKT cells among peripheral blood mononuclear cells in women with early-onset preeclampsia and in healthy pregnant women.

**Table 2:** Peripheral blood mononuclear cell phenotype characteristics in women with early-onset preeclampsia and in healthy pregnant women.

	**Healthy pregnant women**	**Early-onset preeclamptic women**	P-value
CD3+ T cells	67,51±1,5	66,1±4,39	NS
CD4+ T cells	39,98±2,93	37,53±2,98	NS
CD8+ T cells	31,29±2,52	29,92±2,52	NS
Regulatory T cells	0,92±0,12	0,55±0,08	P<0,05
CD3-CD56+ cells	9,91±0,94	8,12±1,25	NS
CD3^-^CD56^dim^cells	8,53±0,92	7,54±1,15	NS
CD3^-^CD56^bright^ cells	1,41±0,12	0,61±0,13	P<0,01
CD3+CD56+ cells	5,11±1,18	3,35±1,11	NS

In both groups, statistical comparisons were made by using the Student’s t-tests. The results were expressed as the mean value±standard error of the mean (SEM). Differences were considered significant when the value of P was equal to or less than 0.05. NS = not statistically significant.

Compared to healthy pregnant controls, in the peripheral blood of early-onset preeclamptic women there is a significant decrease in the frequency of regulatory T cells (0,92±012 vs. 0,55±0,08) and in the frequency of CD56^bright^ cells (1,41±0,12 vs. 0,61±0,13).

### 2: Differential expression of TIM-3 on peripheral blood mononuclear cell subsets in healthy pregnant women

We measured the surface expression of TIM-3 on helper, cytotoxic T cells, NK cells, NK cell subsets and NKT cells by flow cytometry. We observed high surface expression of TIM-3 on most NK cells (median 77,9%) compared with T cells (median 3,7%), cytotoxic (median 13,8%), helper (median 2,2%) T cells or NKT cells (median 5%). CD56^dim^ NK cells are considered mature NK cells and are differentiated from immature CD56^bright^ NK cell subset. We observed that the majority of CD56^dim^ NK cells expressed TIM-3 (median 79,8%), whereas CD56^bright^ NK cells showed a lower level of TIM-3 expression (median 67,6%).

### 3: TIM-3 expression by peripheral blood mononuclear cells in women with early-onset preeclampsia and in healthy pregnant women

**Figure 1:**
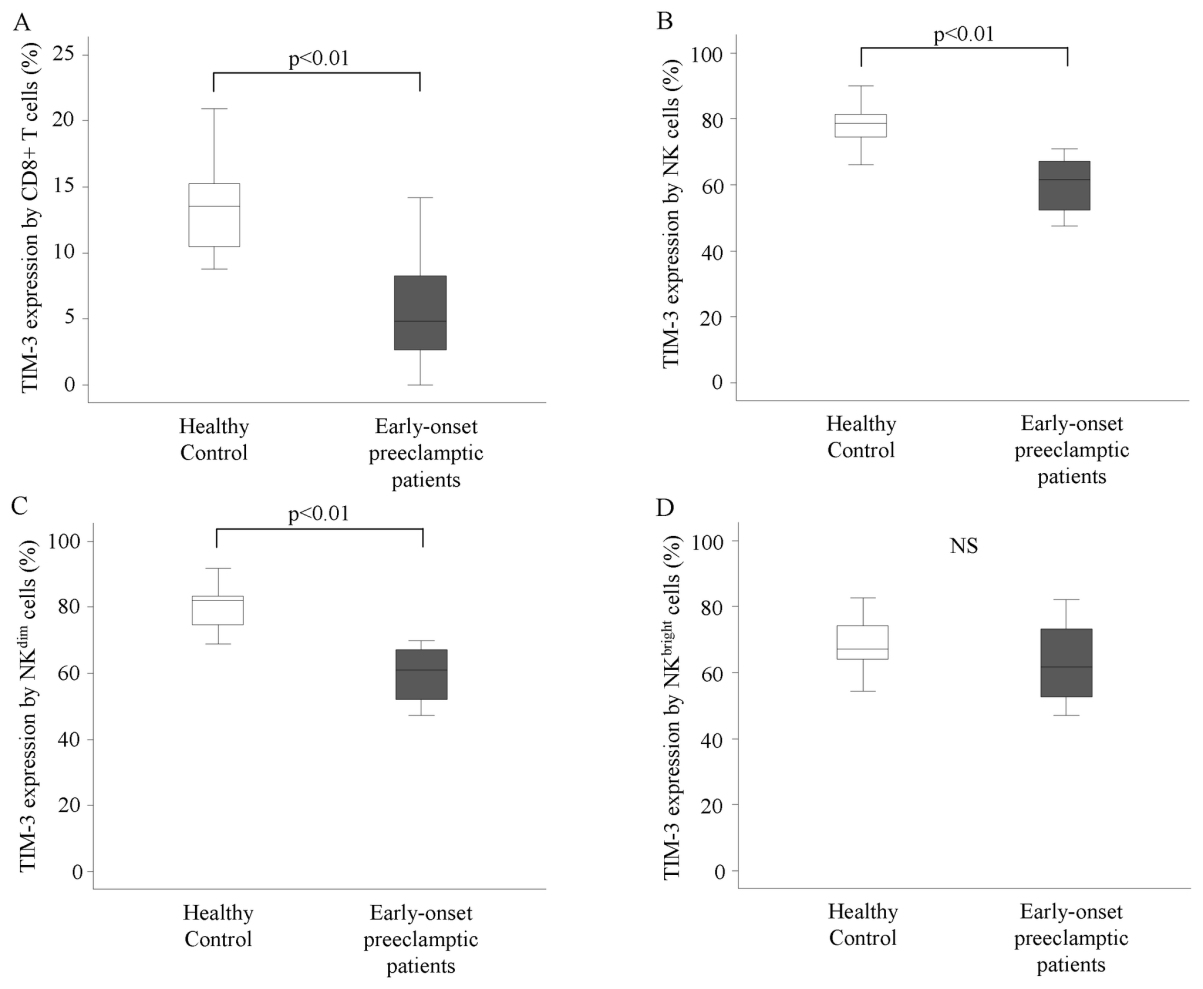
TIM-3 expression by peripheral lymphocytes in women with early-onset preeclampsia and in healthy pregnant women. The expression of TIM-3 by cytotoxic T cells, NK cells, CD56^dim^ NK cells and CD56^bright^ NK cells in the peripheral blood of women with early-onset preeclampsia and in healthy pregnant women. The solid bars represent medians of 10 and 11 determinations, respectively, the boxes indicate the interquartile ranges and the lines show the most extreme observations. Differences were considered statistically significant for P-values ≤0.05. NS = not statistically significant.

Investigating peripheral blood mononuclear cells of women with early-onset preeclampsia, our results showed a decreased TIM-3 expression by T cells, cytotoxic T cells (Figure 1A), NK cells (Figure 1B) and CD56^dim^ NK cells (Figure 1C) compared to healthy pregnant women (T cells: 2,04±0,3 vs. 3,7±0,22; cytotoxic T cells: 7,10±1,78 vs. 13,83±1,17; NK cells: 61,12±2,42 vs. 77,95±2,23; CD56^dim^ NK cells: 60,75±2,4 vs. 79,86±,19).

### 4: Gal-9 expression by peripheral blood mononuclear cells in women with early-onset preeclampsia and in healthy pregnant women

**Figure 2:**
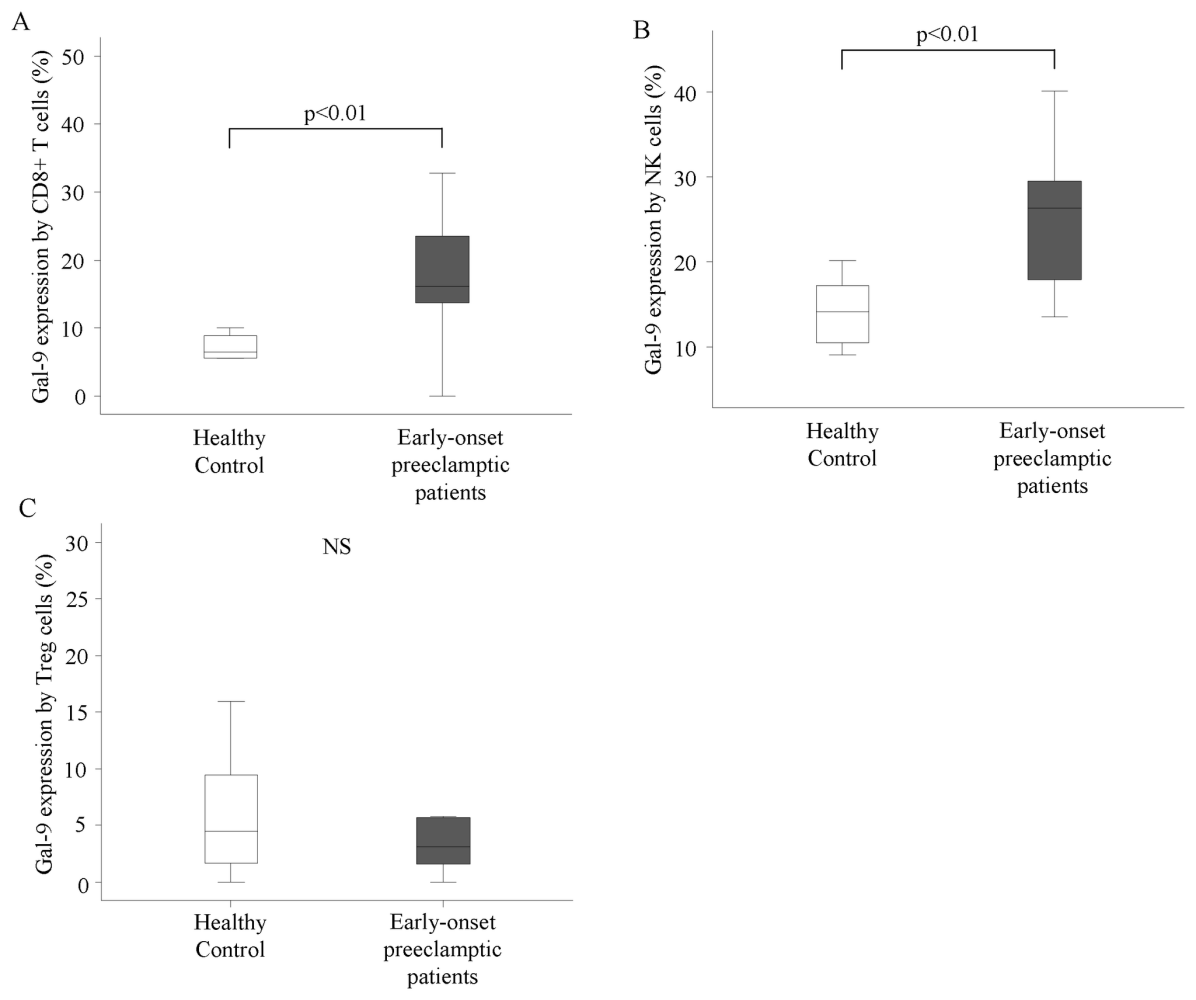
Gal-9 expression by peripheral lymphocytes in women with early-onset preeclampsia and in healthy pregnant women. The expression of Galectin-9 by cytotoxic T cells, NK cells, and regulatory T cells in the peripheral blood of women with early-onset preeclampsia and in healthy pregnant women. The solid bars represent medians of 9 and 11 determinations, respectively, the boxes indicate the interquartile ranges and the lines show the most extreme observations. Differences were considered statistically significant for P-values ≤0.05. NS = not statistically significant.

Analyzing the Gal-9 expression of peripheral lymphocytes we found a notably increased frequency of Gal-9 positive cells in each investigated lymphocyte population and subpopulation (T cells, cytotoxic T cells, NK cells, CD56^dim^ NK cells, CD56^bright^ NK cells and NKT cells) in the case of early-onset preeclamptic patients when compared to healthy pregnant controls (Figure 2A,B). The results are statistical significant in all investigated group except helper T cells, NKT cells and Treg cells (Figure 2C) (T cells: 23,09±3,42 vs. 8,45±1,07; cytotoxic T cells: 20,17±2,87 vs. 8,47±1,17; NK cells: 25,29±2,28 vs. 14,94±1,88; CD56^dim^ NK cells: 25,95±2,49 vs. 16,3±2,1; CD56^bright^ NK cells: 19,36±2,63 vs. 8,26±1,12).

### 5: Cytotoxicity of peripheral cytotoxic T cells and NK cells in women with early-onset preeclampsia and in healthy pregnant women

**Figure 3:**
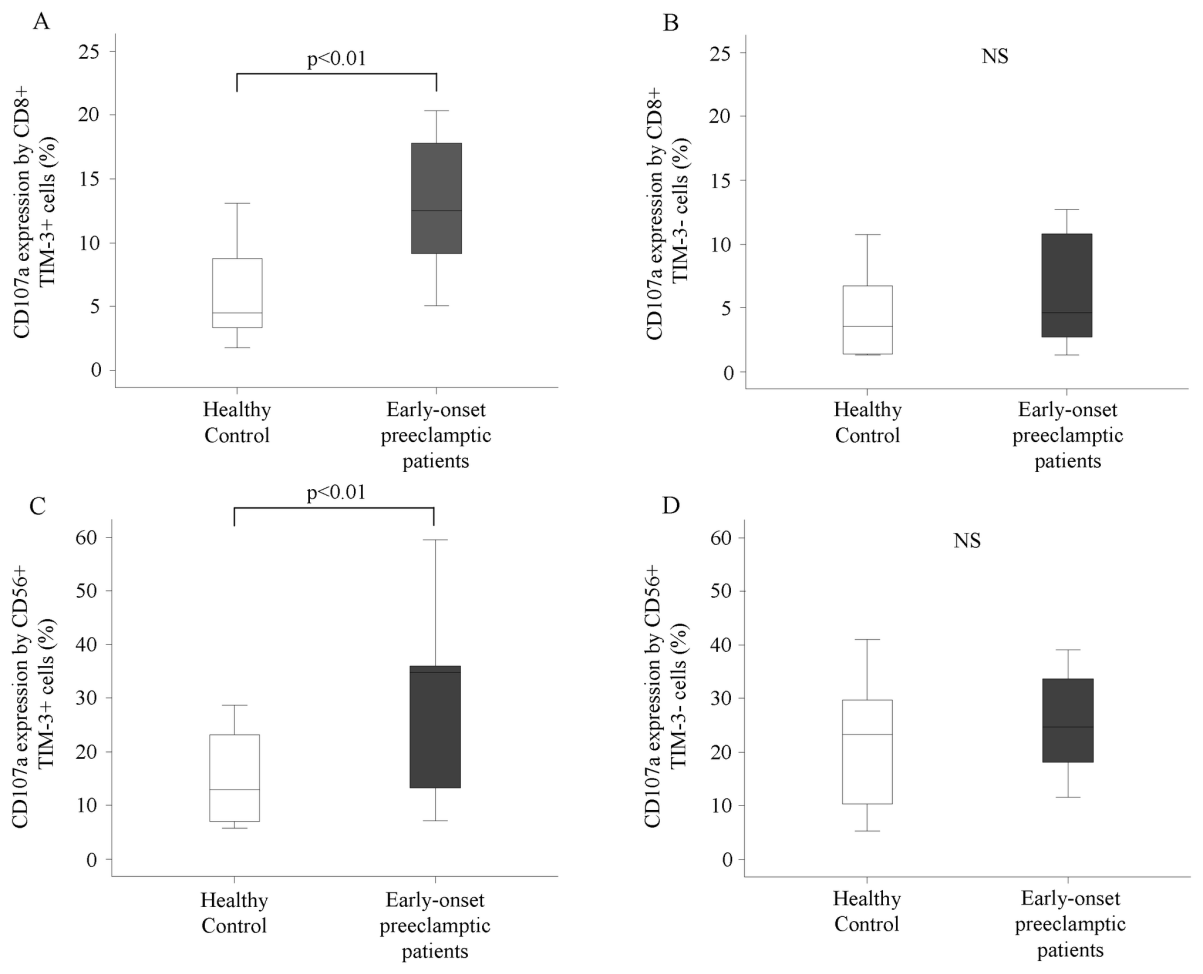
Cytotoxicity of peripheral cytotoxic T and NK cells in women with early-onset preeclampsia and in healthy pregnant women. The expression of CD107a by TIM-3 positive or negative cytotoxic T cells and NK cells in the peripheral blood of women with early-onset preeclampsia and in healthy pregnant women. The solid bars represent medians of 10 and 10 determinations, respectively; the boxes indicate the interquartile ranges and the lines show the most extreme observations. Differences were considered statistically significant for P-values ≤0.05. NS = not statistically significant.

Investigating the cytotoxic activity of cytotoxic T and NK cells, we found that only TIM-3 positive cytotoxic T (Figure 3A,B) and NK cells (Figure 3C,D) showed increased cytotoxicity in women with early-onset preeclampsia compared to healthy pregnant women (cytotoxic T cells: 13,11±1,48 vs. 6,15±1,09; NK cells: 30,02±5,04 vs. 14,32±2,57).

Interestingly, TIM-3 positive CD56^dim^ NK cells from women with early-onset preeclampsia showed significantly increased CD107a expression compared to healthy pregnant women and this difference was not observed in the case of CD56^bright^ NK cells (CD56^dim^ NK cells: 28,53±4,88 vs. 12,68±2,32). Overall, TIM-3 marked the most responsive subset NK cells with respect to degranulation, as marked by CD107a expression.

Analyzing the cytotoxicity of the investigated lymphocyte populations and subpopulations regardless of TIM-3 expression no differences were found between early-onset preeclamptic patients and healthy pregnant donors (data not shown).

## Discussion

The maternal immune system plays a central role in the pathogenesis of preeclampsia, although in different ways, at different sites and with different components in the pre-clinical and clinical stages of the disease. First, there is an immune maladaptation locally to cellular processes of implantation and subsequent trophoblast invasion resulting in a placenta with restricted oxygen and nutrient transfer capacity. In this case cellular lymphoid elements recruited in the decidua are responsible for inadequate operating. These cells are pre-dominantly part of the innate immune system: NK cells, NKT cells, γδ T cells. Maternal symptoms occur when the small sized placenta decompensate, i.e. the result of the placentation disorder becomes the causative agent of the clinical syndrome. At this stage, a systemic non-specific inflammatory response develops induced by placental factors released in the maternal circulation with the involvement of both innate and adaptive immunity. Recognition of placental particles by monocytes and dendritic cells triggers the release of pro-inflammatory cytokines, the key elements for the generalization and exaggeration of inflammatory reactions.

As long as the placenta exists in the uterus the symptoms will persist, even after birth. This fact suggests the failure of immune regulatory mechanism which could dampen long-term inflammatory processes. Several studies confirmed a decreased number and function of regulatory T cells in preeclamptic patients [37]. Treg cells act immunosuppressive by various strategies including inhibitory cytokine secretion (TGF-β, IL-10), direct cytolysis, metabolic disruption and inhibition of dendritic cells [38].

The functional role of Gal-9/TIM-3 pathway was first described as a mechanism to negatively regulate Th1 responses, inhibiting IFN-γ production and inducing cell death. Subsequently, this interaction has been described to have important roles in transplantation immunity, infection, autoimmunity, inflammation and tumor immunity.

Although data about the role of Gal-9/TIM-3 pathway in the pathogenesis of human diseases is emerging, data about their role during human pregnancy is scarce. Our earlier study showed that activated γδ T cells of preeclamptic women have increased cytotoxic potential, which may be due to altered expression of TIM-3 receptor [22]. A recent study by Chabtini et al. showed the possible role of TIM-3-expressing innate immune cells in the regulation of tolerance at the fetomaternal interface using an allogeneic mouse model of pregnancy [39]. The only other human study done by Zhao et al. reported that TIM-3 is up-regulated by monocytes in peripheral blood of pregnant women indicate that abnormal TIM-3 expression level might be connected to the pregnancy loss [40].

These data suggest that Gal-9/TIM-3 pathway could play an important role in the immunoregulation during pregnancy and the altered Galectin-9 and TIM-3 expression could result in an enhanced systemic inflammatory response including the activation of Th1 lymphocytes and type-1 bias in preeclampsia.

In this study we have examined the expression and function of a novel immune receptor TIM-3 in peripheral blood of women with early-onset preeclampsia as our aim is to study the role of TIM-3 positive lymphocyte subpopulations in the maternal syndrome of early-onset preeclampsia with a maternal systemic inflammatory disorder of the second half of pregnancy. During healthy pregnancy NK cells have the highest percentage of cells expressing TIM-3. We observed that the majority of CD56^dim^ NK cells expressed TIM-3, whereas CD56^bright^ NK cells, helper, cytotoxic T cells and NKT cells showed a much lover level of TIM-3 expression. Investigating peripheral blood mononuclear cells of women with early-onset preeclampsia, our results showed a decreased TIM-3 expression by T cells, cytotoxic T cells, NK cells and CD56^dim^ NK cells compared to healthy pregnant women. Interestingly, we found a notably increased frequency of Galectin-9 positive cells in each investigated lymphocyte population and subpopulation (T cells, cytotoxic T cells, NK cells, CD56^dim^ NK cells, CD56^bright^ NK cells and NKT cells) in the case of early-onset preeclamptic patients when compared to healthy pregnant controls.

We further demonstrated increased cytotoxic activity by cytotoxic T and CD56^dim^ NK cells in women with early-onset preeclampsia. One possible mechanistic explanation is that lower expression of surface TIM-3 on immune cells during early-onset preeclampsia may allow cytotoxic T and NK cells to escape Gal-9-induced negative regulation, ultimately leading to uninhibited expansion of Th1 and Th17 response and to persistent inflammatory response usually seen during early-onset preeclampsia. Contrary to these findings however, the percentage of Gal-9 positive effector immune cells (cytotoxic T and NK cells) - except helper and regulatory T cells - were significantly higher in the peripheral blood of patients with preeclampsia than in healthy individuals suggesting a dysfunctional Gal-9 mediated regulatory response.

CD56^bright^ NK cells have been labeled ‘immunoregulatory’ based on their ability to secrete cytokines [41] and home to lymph nodes and tissues and their expansion in humans during states characterized by increased immune tolerance such as healthy pregnancy [42]. Since expanded CD56^bright^ NK cells can limit the survival of activated T cells *in vitro* in a contact-dependent manner [43] we hypothesize that the increased Gal-9 expression by CD56^bright^ NK cells could be a compensatory mechanism as they frequency is significantly decreased during early-onset preeclampsia.

Bielekova et al. published that daclizumab therapy resulted in a gradual expansion of CD56^bright^ NK cells that correlated with the decrease in brain inflammation in multiple sclerosis patients [43,44]. This suggests that daclizumab therapy could facilitate the ability to ultimately modulate Gal-9/TIM-3 pathway for therapeutic benefit.

Further investigations are needed to clear whether the Gal-9-TIM-3 interactions display different immunological responses in healthy pregnant and early-onset preeclamptic women, partially due to the altered expression of these molecules on the cell surface of peripheral lymphocytes.

Anyway, our findings that the strongest cellular cytotoxic response of lymphocytes occurred in the TIM-3 positive subpopulation of different lymphocytes subsets underlines the fact, that the involvement of the immunoregulatory receptor TIM-3 in the pathogenesis of the systemic inflammatory response observed in early-onset preeclampsia is incontestable and represents theoretically a site of therapeutic intervention for immune suppression.
